# Cyclooxygenase (COX)-2 Inhibitors Reduce *Toxoplasma gondii* Infection and Upregulate the Pro-inflammatory Immune Response in *Calomys callosus* Rodents and Human Monocyte Cell Line

**DOI:** 10.3389/fmicb.2019.00225

**Published:** 2019-02-12

**Authors:** Ana Carolina Alcântara Pereira, Rafaela José Silva, Priscila Silva Franco, Angelica de Oliveira Gomes, Guilherme Souza, Iliana Claudia Balga Milian, Mayara Ribeiro, Alessandra Monteiro Rosini, Pâmela Mendonça Guirelli, Eliézer Lucas Pires Ramos, Tiago Wilson Patriarca Mineo, José Roberto Mineo, Neide Maria Silva, Eloisa Amália Vieira Ferro, Bellisa Freitas Barbosa

**Affiliations:** ^1^Laboratory of Immunophysiology of Reproduction, Institute of Biomedical Sciences, Universidade Federal de Uberlândia, Uberlândia, Brazil; ^2^Institute of Natural and Biological Sciences, Universidade Federal do Triângulo Mineiro, Uberaba, Brazil; ^3^Laboratory of Immunoparasitology, Institute of Biomedical Sciences, Universidade Federal de Uberlândia, Uberlândia, Brazil; ^4^Laboratory of Immunopathology, Institute of Biomedical Sciences, Universidade Federal de Uberlândia, Uberlândia, Brazil

**Keywords:** *Toxoplasma gondii*, cyclooxygenase-2, *Calomys callosus*, THP-1 cells, prostaglandin E_2_, immune response

## Abstract

*Toxoplasma gondii* is able to infect a wide range of vertebrates, including humans. Studies show that cyclooxygenase-2 (COX-2) is a modulator of immune response in multiple types of infection, such as *Trypanosoma cruzi*. However, the role of COX-2 during *T. gondii* infection is still unclear. The aim of this study was to investigate the role of COX-2 during infection by moderately or highly virulent strains of *T. gondii* in *Calomys callosus* rodents and human THP-1 cells. *C. callosus* were infected with 50 cysts of *T. gondii* (ME49), treated with COX-2 inhibitors (meloxicam or celecoxib) and evaluated to check body weight and morbidity. After 40 days, brain and serum were collected for detection of *T. gondii* by real-time PCR and immunohistochemistry or cytokines by CBA. Furthermore, peritoneal macrophages or THP-1 cells, infected with RH strain or uninfected, were treated with meloxicam or celecoxib to evaluate the parasite proliferation by colorimetric assay and cytokine production by ELISA. Finally, in order to verify the role of prostaglandin E_2_ in COX-2 mechanism, THP-1 cells were infected, treated with meloxicam or celecoxib plus PGE_2_, and analyzed to parasite proliferation and cytokine production. The data showed that body weight and morbidity of the animals changed after infection by *T. gondii*, under both treatments. Immunohistochemistry and real-time PCR showed a reduction of *T. gondii* in brains of animals treated with both COX-2 inhibitors. Additionally, it was observed that both COX-2 inhibitors controlled the *T. gondii* proliferation in peritoneal macrophages and THP-1 cells, and the treatment with PGE_2_ restored the parasite growth in THP-1 cells blocked to COX-2. In the serum of *Calomys*, upregulation of pro-inflammatory cytokines was detected, while the supernatants of peritoneal macrophages and THP-1 cells demonstrated significant production of TNF and nitrite, or TNF, nitrite and MIF, respectively, under both COX-2 inhibitors. Finally, PGE_2_ treatment in THP-1 cells triggered downmodulation of pro-inflammatory mediators and upregulation of IL-8 and IL-10. Thus, COX-2 is an immune mediator involved in the susceptibility to *T. gondii* regardless of strain or cell types, since inhibition of this enzyme induced control of infection by upregulating important pro-inflammatory mediators against *Toxoplasma*.

## Introduction

Toxoplasmosis is an infectious disease caused by the protozoan parasite *Toxoplasma gondii*, which can invade and replicate in any type of nucleated cell, including macrophages, cells from the nervous system and muscle tissue (Montoya and Liesenfeld, 2004; Dubey, 2010; Robert-Gangneux and Dardé, 2012). Actually, around one-third of the world’s population is infected with *T. gondii*, becoming this parasite one of the best adapted to infect humans (Laliberté and Carruthers, 2008; Dubey, 2010). When the infection is established in immunocompromised patients or during pregnancy, severe manifestations can be observed, especially in newborns or in children congenitally infected, such as retinochoroiditis, encephalitis, and miscarriage (Dubey et al., 2012). A recent study showed that seroprevalence in pregnant women in Brazil is 36–92%, so characterizing a serious public health problem (Foroutan-Rad et al., 2016). Furthermore, programs of prevention of congenital toxoplasmosis in pregnant women can be only found in a limited number of countries. A French screening program established since 1992 requires monthly testes in pregnant women until delivery, in order to reduce significantly the rate of congenital transmission (Peyron et al., 2017). Thus, new strategies to prevent or treat the toxoplasmosis are welcome, in order to minimize the public health problem and to improve the quality of life of children and immunocompromised individuals infected by the parasite.

The immune response to *T. gondii* infection is predominantly pro-inflammatory (Lang et al., 2007). During infection, cells from innate immunity, such as macrophages, neutrophils, and dendritic cells recognize the parasite by pathogen-associated molecular patterns (Hou et al., 2011; Koblansky et al., 2013; Gorfu et al., 2014) and produce high levels of pro-inflammatory cytokines, such as interleukin (IL)-12, which activates CD4^+^ T lymphocytes to produce interferon (IFN)-γ, the major cytokine involved in control of *T. gondii* (Gazzinelli et al., 1994; Kemp et al., 2013; Koblansky et al., 2013; Behnke et al., 2017). In parallel to IFN-γ, other pro-inflammatory cytokines, such as IL-6, tumoral necrosis factor (TNF), IL-17A, IL-2 and macrophage migration inhibitory factor (MIF) also participate significantly in the immunity against *T. gondii* (Kelly et al., 2005; Castro et al., 2013; Barbosa et al., 2014, 2015; Gomes et al., 2018). Our previous studies demonstrated that human trophoblast cells controlled *T. gondii* intracellular proliferation in a MIF-dose-dependent manner, since only high concentrations of recombinant MIF (rMIF) were able to reduce the parasite growth. On the other hand, low concentrations of rMIF triggered significant production of prostaglandin E_2_ (PGE_2_) and, consequently, increased susceptibility to *T. gondii* in human trophoblast cells, showing the potential effect of PGE_2_ to favor parasite replication (Barbosa et al., 2014). Thus, the parasite can use some molecules from the host, such as PGE_2_, to evade the immune response and to establish definitely into the host cells (Barbosa et al., 2014).

Prostaglandins are lipid mediators involved in many activities, including inflammatory and immunological functions, since the participation of prostaglandins in the cellular activation and maturation, and cytokine production in cells from innate immunity as macrophages and dendritic cells, has been confirmed (Nagamatsu and Schust, 2010; Kalinski, 2012). Prostaglandins, especially PGE_2_, are synthesized when phospholipase A_2_ promotes the release of arachidonic acid from the plasmatic membrane (Pawlowski et al., 1983; Agard et al., 2013). Subsequently, the arachidonic acid is converted into prostaglandins by enzymes called cyclooxygenases (COXs). There are at least two isoforms of COX: COX-1, constitutively expressed in all cell types, and COX-2, which is induced by inflammatory mediators, mainly cytokines (Batlouni, 2010; Agard et al., 2013; Sharma et al., 2017; Martínez-Colón and Moore, 2018).

Many studies demonstrate the role of COX-2 and PGE_2_ during infection triggered by *Trypanosoma cruzi*, and these mediators are shown to be essential to survival of this pathogen in the host cells, inducing replication and dissemination of the pathogen, as well as downmodulation of immune response. COX-2 expression is significantly increased when infection by *T. cruzi* is present, confirming that this parasite is a potent inductor of COX-2 (Moraes et al., 2015). Mice infected with *T. cruzi* showed reduced parasitism in blood and cardiac muscle when treated with COX-2 inhibitors (meloxicam, etoricoxib, sodium salicylate, aspirin, or celecoxib) (Michelin et al., 2005; Abdalla et al., 2008; Tatakihara et al., 2008). In addition, COX inhibitors diminished the internalization of *T. cruzi* in mice peritoneal macrophages and, at the same time, upregulated IL-1β and nitrite, demonstrating the potential role of COX in favoring the infection by *T. cruzi* by downmodulating pro-inflammatory mediators (Malvezi et al., 2014). Thus, the roles of COX-2 and PGE_2_ during infections triggered by *T. cruzi* are already widely discussed in the literature, since both are mentioned as inductors of the immunosuppression observed during the acute phase of Chagas disease, favoring the persistence of the parasite into host cells (Pinge-Filho et al., 1999).

However, the role of COX-2 during infections triggered by *T. gondii* is still unclear. Mota et al. (2014) observed an increase of lipid droplets in mice peritoneal macrophages infected with *T. gondii*, and it was associated with high levels of PGE_2_ and low levels of nitrite. Furthermore, primary cultures of skeletal muscle cells infected with *T. gondii* also presented higher numbers of lipid droplets and increased COX-2 and PGE_2_ expression (Gomes et al., 2014), but there are no studies demonstrating an association between COX-2 and susceptibility to *T. gondii* infection with different strains of this parasite. In this sense, the aim of the present study was to investigate the role of COX-2 during infection by moderately or highly virulent strains of *T. gondii* in *Calomys callosus*, a rodent of the family Cricetidae, widely distributed in Central Brazil. Our research group has been used *C. callosus* widely as an experimental model for toxoplasmosis studies (Barbosa et al., 2007, 2012; Costa et al., 2009; Franco et al., 2014, 2015), since this rodent has been described as a reservoir for various infectious agents (Borges et al., 1992; Favoreto-Júnior et al., 1998). Also, we verified the role of COX-2 during infection by *T. gondii* in human monocyte cell line (THP-1 cells), in order to compare the COX-2 activity between rodent cells and human cells.

## Materials and Methods

### Animals

*Calomys callosus* (8 to 10 weeks) were kept in a room with adequate temperature (25 ± 2°C) and luminosity (12-h light, 12-h dark), under specific pathogen-free conditions, and free access to food and water. The colony was maintained in the Animal Experimentation Center of the Institute of Biomedical Sciences, Universidade Federal de Uberlândia, Uberlândia, Brazil. This study was carried out in accordance with the recommendations of institutional guidelines for animal ethics, Comissão de Ética na Utilização de Animais (CEUA-UFU). All procedures were approved and conducted according to institutional guidelines for animal ethics (Approval No. 232/13).

### Cell Culture

Human monocyte (THP-1) and human choriocarcinoma (BeWo) cell lines were obtained from American Type Culture Collection (ATCC, Manassas, VA, United States). Both cells were cultured in culture flasks of 75 cm^2^ containing Roswell Park Memorial Institute (RPMI) 1640 medium (Cultilab, Campinas, Brazil) with penicillin (100 U/mL) and streptomycin (100 μg/mL) antibiotics (both from Sigma Chemical Co., St. Louis, MO, United States), and 10% heat-inactivated fetal bovine serum (FBS) (Cultilab). The cells were maintained in a humidified incubator at 37°C and 5% CO_2_ (Castro et al., 2013; Barbosa et al., 2015).

### Parasite Strains

The *T. gondii* ME49 strain (moderately virulent – type II) was obtained from previously infected *C. callosus* males (Barbosa et al., 2007). Briefly, the males were euthanized and the brains were collected. Next, the brains were washed in sterile 0.01 M phosphate-buffered saline (PBS, pH 7.2) to remove the excess blood, fragmented with a syringe and a needle (25 × 7 gauge) for 5 min at least, and again washed with PBS by centrifugation at 1000 × *g* for 10 min. Finally, 20 μL homogenized brains were placed in glass slides under coverslip and the number of cysts were counted with light microscopy (Barbosa et al., 2007).

*Toxoplasma gondii* tachyzoites of the 2F1 clone were provided from Dr. Vern Carruthers (Medical School of Michigan University, Ann Arbor, MI, United States). This clone is derived from RH parasites, a highly virulent strain from type I. These tachyzoites constitutively express cytoplasmic beta-galactosidase, which enable a colorimetric assay to measure the parasites in cells or tissues (Castro et al., 2013; Barbosa et al., 2015). The parasites were propagated *in vitro* in BeWo cells cultured in RPMI 1640 medium containing the penicillin and streptomycin antibiotics, as above described, and 2% FBS.

### Treatment of *C. callosus* Females With COX-2 Inhibitors

In the first step of the experiments, we investigated the role of COX-2 inhibitors during the chronic phase of *T. gondii* infection.

For this purpose, *C. callosus* females (*n* = 24) were analyzed for body weight and morbidity score, and immediately infected with 50 cysts of *T. gondii* (ME49 strain) via oral route (day 1). Twenty-four hours later (day 2), the females were divided into three groups with eight animals, as follows: females infected and treated with water (group 1 – control); females infected and treated with a preferential COX-2 inhibitor, meloxicam (Eurofarma Laboratórios, São Paulo, Brazil) at 0.5 mg/kg (Abdalla et al., 2008) (group 2); and females infected and treated with a specific COX-2 inhibitor, celecoxib (Pfizer Pharmaceuticals LLC, Guarulhos, Brazil) at 5 mg/kg (Short et al., 2013) (group 3). All the groups received the treatments daily, diluted in filtered drinking water, via oral route, for 40 consecutive days. In these 40 days, the animals were monitored daily to observe any signal of suffering and, in positive case, the euthanasia would be carried out immediately. The body weight change and score morbidity were evaluated every 72 h. Morbidity was assessed based on the clinical parameters as previously described, with minor modifications: sleek/glossy coat, bright and active (score 0); hunched, starry stiff coat (score 1); and reluctance to move (score 2) (Bartley et al., 2006; Franco et al., 2014).

After 40 days of treatment and infection (day 42), the females were anesthetized, the blood was collected and serum stored at −80°C for posterior cytokine and nitrite measurements. Next, the females were euthanized and the brain was collected for further detection of tissue parasitism by quantitative real-time PCR and immunohistochemistry, as well as Western blotting for COX-2 expression. Uninfected and untreated females (*n* = 8) were used in the experiments only to obtain serum and brain in order to detect cytokine and nitrite or COX-2 expression to compare with infected and untreated animals.

### DNA Extraction and Quantitative Real-Time PCR (qRT-PCR)

The *T. gondii* DNA was quantified by RT-PCR and it was carried out according to our previous study (Franco et al., 2014), with minor modifications. The brains from *C. callosus* were frozen in liquid nitrogen and immediately processed to extract total DNA using the Promega Wizard Genomic DNA Purification kit following the manufacturer’s instructions (Promega Co., Madison, WI, United States). DNA for analyze was extracted from 20 mg of the tissues. The quantification was performed using UV spectrophotometry (ND1000 Spectrophotometer; NanoDrop Technologies, Wilmington, DE, United States). The samples were prepared with Promega Master Mix Kit Go Taq^®^ Quantitative Real-Time PCR, and the StepOnePlus^®^ Real-Time PCR System (Applied Biosystems, Carlsbad, CA, United States) was used to thermal profile of the reaction stages. The primer pairs used were (Tg529 forward, 5′-GCTCCTCCAGCCGTCTTC-3′; and Tg529 reverse, 5′-CCTCACCCTCGCCTTCAT-3′) (Exxtend, Campinas, Brazil), amplifying a region with 529 base pairs (bp), as described by Homan et al. (2000). Positive control of the reaction was a suspension of RH strain tachyzoites (10^4^ tachyzoites/mL), and ultrapure water was used as negative control. The standard curve and samples were carried out with 100 and 200 ng of DNA targets, respectively.

### Immunohistochemistry for *T. gondii* Detection

To verify the presence of *T. gondii* cysts in brains of *C. callosus*, the samples were submitted to immunohistochemistry assay. For this purpose, the brains were fixed in 10% formalin in PBS for 24 h, embedded in paraffin and the glass slides containing sections of 4 μm obtained in microtome were treated for 5 min with citric acid (pH 6.0) for antigenic recovery. Next, 5% acetic acid solution was added on the sections for 8 min to avoid detection of endogenous phosphatase, and posteriorly the sections were treated with 2.5% goat serum for 45 min at 37°C to inhibit recognition of non-specific sites. After, the sections were incubated with 1:100 primary antibody (previously infected *C. callosus* serum) during 12 h at 4°C, washed in Tris-buffered saline solution (TBS, pH 7.4) to remove the excess of antibodies, and treated with 1:600 secondary antibody (biotinylated goat-anti mouse IgG, Jackson Immuno Research Laboratories, West Grove, PA, United States) for 1 h at 37°C. Finally, the detection of parasites was developed for 10 min with fast red naphthol (Sigma) at room temperature, and the brains counterstained with Harris’s hematoxylin were visualized under light microscope (BX40, Olympus, Tokyo, Japan) (Gomes et al., 2011; Castro-Filice et al., 2014).

### Culture of Peritoneal Macrophages

Peritoneal macrophages were obtained from 10 to 12-week old female *C. callosus*, according to Rodrigues et al. (2012) with minor modifications. Briefly, 3 days before collecting peritoneal macrophages, females (*n* = 6) were stimulated with 1 mL of 4% thioglycollate medium (BD Bioscience, San Jose, CA, United States) to induce recruitment of macrophages. Next, the animals were euthanized and the peritoneum washed with 5 mL ice cold RPMI 1640 medium supplemented with penicillin (100 U/mL), streptomycin (100 μg/mL), and 10% FBS. The cells were harvested, centrifuged at 400 × *g* for 5 min, adjusted to 1 × 10^5^ cells/200 μL in RPMI supplemented with penicillin (100 U/mL), streptomycin (100 μg/mL) and 10% FBS, seeded in 96-well plates (Corning Corporation, Cambridge, MA, United States) and incubated in a humidified incubator at 37°C and 5% CO_2_. After 24 h, the supernatants were collected to remove the non-adherent cells. The adherent cells were considered peritoneal macrophages (Rodrigues et al., 2012).

### Viability Assay in Peritoneal Macrophages Treated With COX-2 Inhibitors

The peritoneal macrophages (1 × 10^5^ cells/200 μL in 96-well plates) were treated with increasing concentrations of meloxicam or celecoxib (1, 5, 10, 250, 500, and 1000 μg/mL) in RPMI supplemented with penicillin, streptomycin and 10% FBS for 24 h in a humidified incubator at 37°C and 5% CO_2_. As control, the cells were treated with medium only. For viability assay, the cells were incubated for 4 h in RPMI medium containing 3-(4,5-Dimethyl-2-thiazolyl)-2,5-diphenyl-2H-tetrazolium bromide (MTT) at 37°C and 5% CO_2_. Next, the formazan crystals produced by viable cells were homogenized for 30 min in 10% sodium dodecyl sulfate (SDS, Sigma) and 50% *N*,*N*-dimethyl formamide (Sigma) (Mosmann, 1983), and the optical densities measured at 570 nm (Titertek Multiskan Plus, Flow Laboratories, McLean, VA, United States). The optical densities of all experimental conditions with meloxicam or celecoxib were calculated to percentage in relation to untreated cells, considered as 100% viability. Two independent experiments with six replicates were performed.

### *T. gondii* Intracellular Proliferation in Peritoneal Macrophages Treated With COX-2 Inhibitors

After verifying the toxicity of meloxicam or celecoxib by MTT, we chose the non-toxic concentrations of both inhibitors to analyze the *T. gondii* intracellular proliferation in peritoneal macrophages. For this purpose, peritoneal macrophages (1 × 10^5^ cells/200 μL in 96-well plates) were infected with *T. gondii* tachyzoites (2F1 clone, RH strain) in a proportion of three parasites per cell (3:1), and after 3 h of culture, the cells were treated with 1, 5, 10, 250, 500, or 1000 μg/mL meloxicam or 1, 5, or 10 μg/mL celecoxib in RPMI supplemented with penicillin, streptomycin and 10% FBS for an additional 24 h. As controls, macrophages were treated and not infected, infected and not treated, or not infected and not treated. Then, the supernatants were collected and stored at −80°C for posterior cytokine and nitrite measures, while the cells were submitted to *T. gondii* intracellular proliferation assay by β-galactosidase colorimetric reaction as previously described (Barbosa et al., 2015). *T. gondii* intracellular proliferation was presented as number of tachyzoites and calculated in reference to a standard curve obtained from free 2F1 parasites (detection limit: 15.6 × 10^3^ tachyzoites) (Barbosa et al., 2015). Three independent experiments with six replicates were performed.

### *T. gondii* Intracellular Proliferation in THP-1 Cells Treated With COX-2 Inhibitors and/or PGE_2_

In order to verify the COX-2 intracellular mechanism in human cells, besides tissue and macrophage of rodents, THP-1 cells (3 × 10^4^ cells/200 μL in 96-well plates) were infected with 2F1 *T. gondii* tachyzoites (3:1) and treated with 250 μg/mL meloxicam or 10 μg/mL celecoxib, and incubated in the same culture conditions, as above described, for an additional 24 h.

After, to evaluate the role of PGE_2_ and association between COX-2 and PGE_2_ during infection with *T. gondii*, THP-1 cells (3 × 10^4^ cells/200 μL in 96-well plates) were infected with 2F1 tachyzoites (3:1), treated with several concentrations of PGE_2_ (Cayman Chemical, Ann Arbor, MI, United States) (0.015, 0.150, 8, 20, 40, or 80 ng/mL) according to our previous study (Barbosa et al., 2014), and maintained in culture for additional 24 h. In a second step, THP-1 cells (3 × 10^4^ cells/200 μL in 96-well plates) were infected (3:1), and treated with meloxicam (250 μg/mL) or celecoxib (10 μg/mL) plus 0.150 or 80 ng/mL PGE_2_ for 24 h. As controls, THP-1 cells were treated and not infected, infected and not treated, or not infected and not treated. Also, THP-1 cells were infected and treated with 0.022% DMSO, the diluent of PGE_2_, to exclude any effect of DMSO in the parasitism or cytokine/nitrite production.

Supernatants were collected and stored at −80°C for posterior cytokine and nitrite measures, while the cells were submitted to *T. gondii* intracellular proliferation assay by β-galactosidase colorimetric reaction as previously described (Barbosa et al., 2015). Two independent experiments with eight replicates were performed.

### Western Blotting

In order to confirm the inhibition or reduction of COX-2 expression in cells (peritoneal macrophages or THP-1 cells) and brains of females treated with meloxicam or celecoxib, we performed Western blotting to detect COX-2.

For this purpose, peritoneal macrophages or THP-1 cells (1 × 10^6^ cells/2000 μL in 6-well plates) were infected (3:1) and treated with meloxicam (250, 500, and 1000 μg/mL for peritoneal macrophages, or 250 μg/mL for THP-1) or celecoxib (1, 5, or 10 μg/mL for peritoneal macrophages, or 10 μg/mL for THP-1) in RPMI medium containing 10% FBS during 24 h. As positive control for COX-2 expression, peritoneal macrophages were not infected and only treated with 1 μg/mL LPS (InvivoGen, Toulouse, France) for 24 h. After, the cells were collected, lysed in ice RIPA buffer [50 mM Tris-HCl, 150 mM NaCl, 1% Triton X-100, 1% (w/v) sodium deoxycholate, and 0.1% (w/v) sodium dodecyl sulfate (SDS), pH 7.5] supplemented with protease inhibitor cocktail (Complete, Roche Diagnostic, Mannheim, Germany), 1 mM sodium orthovanadate (Na_3_VO_4_) and sodium fluoride (NaF) (Sigma), and submitted to three freeze-thaw cycles for protein extraction. The total protein was centrifuged (21,000 × *g*, 10 min, 4°C), measured by Bradford assay (Bradford, 1976), and submitted to Western blotting for COX-2 detection. In parallel, brains (fragments) of *C. callosus* females from all experimental groups were lysed as above described and submitted to Western blotting for COX-2.

Electrophoresis in polyacrylamide gel (SDS-PAGE) was performed in 100 μg total protein. Next, the proteins were transferred to polyvinylidene fluoride (PVDF) membranes (Thermo Scientific, Rockford, IL, United States), blocked with 4% skimmed milk in Tris-buffered saline solution (TBS: 25 mM TRIS and 0.15 M NaCl, pH 7.4) for 1 h, and incubated overnight with goat polyclonal anti-COX-2 (1:100, R&D Systems, Minneapolis, MN, United States) or mouse monoclonal anti-beta-actin (1:1000, Santa Cruz Biotechnology, Santa Cruz, CA, United States) in TBS. Then, membranes were incubated with respective HRP-secondary antibodies (1:3000, Jackson ImmunoResearch Laboratories) in TBS with 2% skimmed milk for 2 h, washed and revealed by chemiluminescence kit (Thermo Scientific) in ChemiDoc MP Imaging System (BIO-RAD Laboratories, Inc., Hercules, CA, United States).

### Measurement of Cytokines and Nitrite

The serums of *C. callosus* from three experimental groups were used for the detection of Th1, Th2, and Th17 cytokines by cytometric bead array (CBA). Mouse cytokines (IL-2, IL-4, IL-6, IFN-γ, TNF, IL-17A, and IL-10) were measured in serums using the Th1/Th2/Th17 kit according to the BD Bioscience instructions. The level of cytokines was evaluated under FACSCalibur flow cytometry (BD Company, San Diego, CA, United States), processed with BD Cell Quest and CBA softwares, and the data were presented as mean fluorescence intensity (MFI) according to a standard curve of each cytokine (Franco et al., 2011; Barbosa et al., 2015).

In addition, the supernatants of cell cultures were used to measure IL-6, TNF, IFN-γ, IL-17A, and IL-10 cytokines in peritoneal macrophages, or IL-6, TNF, IFN-γ, IL-10, TGF-β1, MIF, and IL-8 cytokines in THP-1 cells, by sandwich ELISA according to the BD Bioscience or R&D Systems instructions. The data were expressed in pg/mL according to a standard curve of each cytokine.

Both serums and supernatants were used to measure nitrite by the Griess method (Green et al., 1982). Briefly, serum and supernatant were added to 96-well plates, incubated with Griess reagent (1% sulfanilamide dihydrochloride and 0.1% naphthylenediamide dihydrochloride in 2.5% H_3_PO_4_) for 10–20 min, and the absorbance was read in a plate reader (Titertek Multiskan Plus) at 570 nm. The nitrite concentration was determined with reference to a standard curve of sodium nitrite (μM/mL).

### Statistical Analysis

Statistical analysis between different experimental conditions were verified by Mann–Whitney test or Bonferroni multiple comparison (one-way or two-way ANOVA) using GraphPad Prisma version 5.0 (GraphPad Software, Inc., San Diego, CA, United States). *P <* 0.05 was considered as significant difference.

## Results

### *T. gondii* Infection in *C. callosus* Induced Body Weight Change and Moderate Clinical Signs, Regardless of COX-2 Inhibitor Treatment

In a first stage, we performed experiments during the chronic phase of infection. Body weight change and morbidity scores were verified every 72 h in *C. callosus* females infected with *T. gondii* (ME49 strain) and treated with COX-2 inhibitors (meloxicam or celecoxib) for 40 consecutive days. It is important to emphasize that *C. callosus* has a mean body weight around 40 g, ME49 strain does not cause severe disease in these animals, and *Calomys* are widely used as toxoplasmosis model study (Ferro et al., 2002; Barbosa et al., 2007, 2012; Franco et al., 2014, 2015). The mortality rate of these animals is generally very low after *T. gondii* infection with moderately virulent strains, which enable to monitoring for many days. Thus, no animal from the experimental conditions tested succumbed to infection. In addition, no signal of severe suffering was observed in animals, then euthanasia was not performed before 40 days of infection/treatment as an endpoint strategy.

**FIGURE 1:**
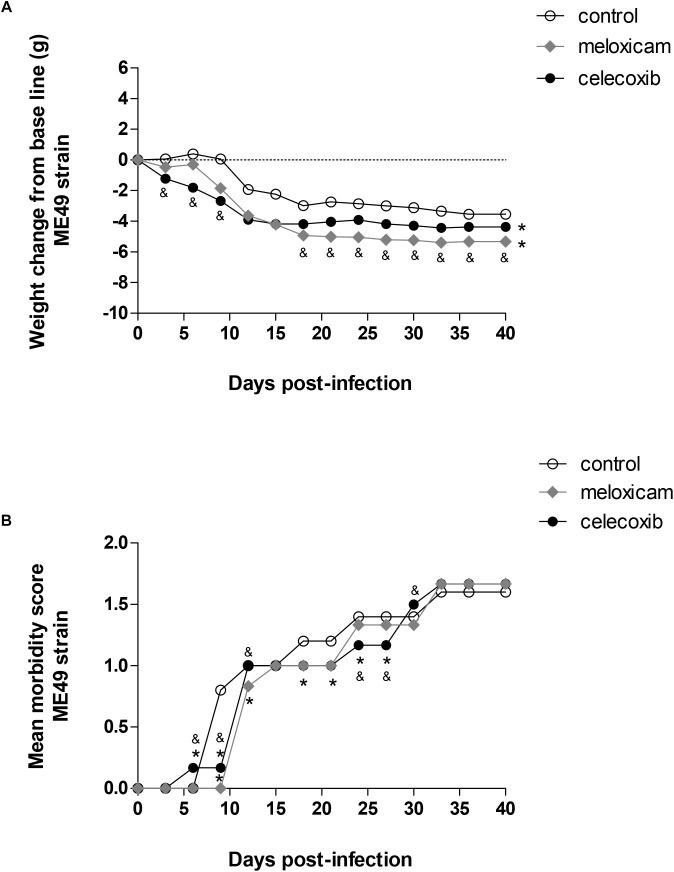
Body weight change and score morbidity. *Calomys callosus* females were infected orally with 50 cysts of *Toxoplasma gondii* (ME49 strain), treated or untreated with meloxicam (0.5 mg/kg) or celecoxib (5 mg/kg) daily and monitored for body-weight change **(A)** and morbidity score **(B)** for 40 days. Dotted lines represent the absence of body-weight change **(A)**. Differences between groups were verified by Mann–Whitney test. Significant differences from day 3 post-infection (p.i.) to 39 p.i. in relation to untreated/infected animals (control) (^∗^*P* < 0.05), and celecoxib/infected animals (^&^*P* < 0.05).

In general, changes in body weight and moderate clinical signs were induced by *T. gondii* in *C. callosus*, regardless of COX-2 inhibitor treatment (Figure 1).

All the COX-2 inhibitors, celecoxib (*P* < 0.001) and meloxicam (*P* < 0.001), induced significant body weight loss from day 3 post-infection (p.i.) to 39 p.i. in comparison to untreated females (control). It means that significant body weight loss was detected during all days evaluated in animals treated with meloxicam or celecoxib (*P* < 0.001), regardless of time of infection (Figure 1A). In addition, celecoxib-treated females lost more weight than meloxicam-treated females until the day 9 p.i. (*P* < 0.0001) (Figure 1A). However, from day 18 p.i. onwards, meloxicam-treated females started to lose weight more significantly compared to celecoxib-treated females (*P* < 0.0001) (Figure 1A).

Females treated with celecoxib were the first group to present clinical signs, showing increase of morbidity score at 6 days p.i. (*P* < 0.0001), since the animals started to present slight hunched, starry stiff coat (Figure 1B). On the other hand, from day 9 to 27 p.i., except at days 12 and 15, untreated females (control) started to present more significant clinical signs in relation to animals treated with COX-2 inhibitors (*P* < 0.0001), showing increased hunched, starry stiff coat and, at the same time, the females started to present moderately slower locomotion and reactions (Figure 1B). At 6, 9, 12, and 30 days p.i., morbidity score was higher in females treated with celecoxib (progressive increase of hunched, starry stiff coat and lower ability to move) in comparison to meloxicam-treated females (*P* < 0.0001) (Figure 1B). However, at 24 and 27 days p.i., females treated with meloxicam demonstrated higher morbidity score (hunched, starry stiff coat and lower ability to move) compared to celecoxib-treated females (*P* < 0.0001) (Figure 1B). From days 33 to 39 p.i., all the animals demonstrated similar morbidity score (increased hunched, starry stiff coat and lower ability to move) (Figure 1B).

### COX-2 Inhibitors Reduced Significantly the Tissue Parasitism in *C. callosus* Infected With *T. gondii* ME49 Strain

**FIGURE 2:**
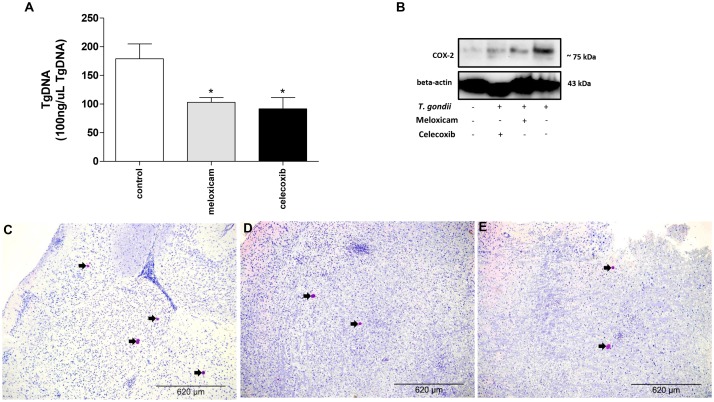
Parasite burden and COX-2 expression in *C. callosus* after infection with *T. gondii* and treatment with COX-2 inhibitors. Females were infected orally with 50 cysts of *T. gondii* (ME49 strain), treated or untreated with meloxicam (0.5 mg/kg) or celecoxib (5 mg/kg) daily, euthanized after 40 days of infection and treatment, and the brains collected and analyzed by real-time PCR to quantify the parasite burden **(A)** or Western blotting to detect COX-2 and beta-actin expressions **(B)**. The data are shown as *T. gondii* DNA (TgDNA) concentration in 100 ng/μL total DNA **(A)**. Differences between groups were analyzed by one-way ANOVA with the Bonferroni multiple comparison *post hoc* test. Significant differences in relation to untreated/infected animals (control) (^∗^*P* < 0.05) **(A)**. Representative photomicrographs of brains of *C. callosus* infected and untreated (control) **(C)**, infected and treated with meloxicam **(D)** or celecoxib **(E)**. Immunohistochemical sections counterstained by Harris’s hematoxylin. Arrows indicate parasites inside the cyst-like structures. Bar scale: 620 μm.

After verifying the clinical parameters, we investigated the parasitism in brains of infected females treated or untreated with COX-2 inhibitors for 40 consecutive days (Figure 2).

Females treated with meloxicam or celecoxib showed lower parasite burdens in the brain in comparison to untreated and infected animals (control) (*P* = 0.0380) (Figure 2A). In order to confirm the reduction of COX-2 expression in these *C. callosus* females treated with COX-2 inhibitors, Western blotting was performed. As results, brains of uninfected and untreated females presented basal levels of COX-2. In the presence of *T. gondii* infection, untreated animals showed upregulation of COX-2, but when females were treated with meloxicam or celecoxib, a reduction of COX-2 in tissue brain was detected when compared to untreated and infected females (Figure 2B). In addition, we quantified the number of cyst-like structures per brain tissue section (around 40 fields per section) using immunohistochemistry. It was verified a lower number of cyst-like structures in brains of females treated with meloxicam (mean of 18 structures) or celecoxib (mean of 15 structures) if compared to untreated females (mean of 37 structures) (data not shown). Representative photomicrographs of immunohistochemistry showing the presence of *T. gondii* in the brain, where it is possible to observe a reduced number of cyst-like structures (arrowed) in animals treated with meloxicam (Figure 2D) or celecoxib (Figure 2E) compared to untreated animals (Figure 2C).

Thus, it is possible to conclude that COX-2 is an important mediator during the chronic phase of *T. gondii* infection.

### COX-2 Inhibitors Upregulated Pro-inflammatory Cytokines in *C. callosus* Infected With *T. gondii* ME49 Strain

**FIGURE 3:**
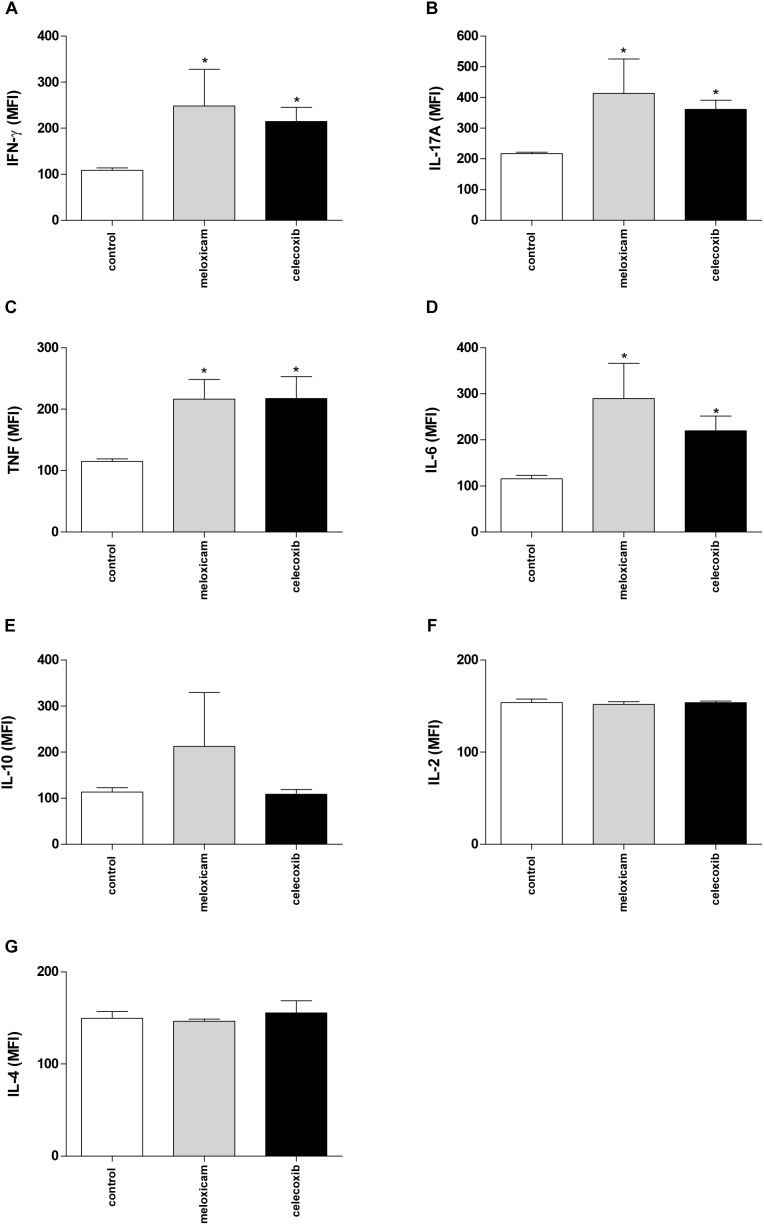
Cytokine production in *C. callosus* after infection with *T. gondii* and treatment with COX-2 inhibitors. Females were infected orally with 50 cysts of *T. gondii* (ME49 strain), and treated or untreated with meloxicam (0.5 mg/kg) or celecoxib (5 mg/kg) daily for 40 days. The serum was collected for measurement of IFN-γ **(A)**, IL-17A **(B)**, TNF **(C)**, IL-6 **(D)**, IL-10 **(E)**, IL-2 **(F)**, and IL-4 **(G)** by CBA. Data are expressed as MFI according to the standard curve. Differences between groups were analyzed by one-way ANOVA with the Bonferroni multiple comparison *post hoc* test. Significant differences in relation to untreated/infected animals (control) (^∗^*P* < 0.05).

After evaluating the parasite burden in tissues of infected *C. callosus*, we verified the cytokine levels in serum from those infected animals treated or untreated with COX-2 inhibitors for 40 consecutive days (Figure 3).

Untreated and infected females (control) showed production of all cytokines measured (Figure 3A–G). However, females treated with meloxicam or celecoxib demonstrated upregulation of IFN-γ (*P* = 0.049), IL-17A (*P* = 0.0115), TNF (*P* = 0.0289), and IL-6 (*P* = 0.0321), when compared to untreated animals (Figure 3A–D). No significant differences between untreated and treated females were detected on IL-10, IL-2 or IL-4 levels (Figure 3E–G). The nitrite production was undetected in all conditions, and untreated/uninfected females showed undetected levels of cytokines (data not shown).

### COX-2 Inhibitors Reduced Significantly the *T. gondii* Intracellular Proliferation in Peritoneal Macrophages Infected With RH Strain

In order to verify the effects of COX-2 inhibitors on infection by *T. gondii* triggered by a highly virulent strain, we performed experiments using peritoneal macrophages of *C. callosus*. For this purpose, we cultured these macrophages *in vitro*, treated with COX-2 inhibitors and infected with *T. gondii* RH (clone 2F1) strain.

**FIGURE 4:**
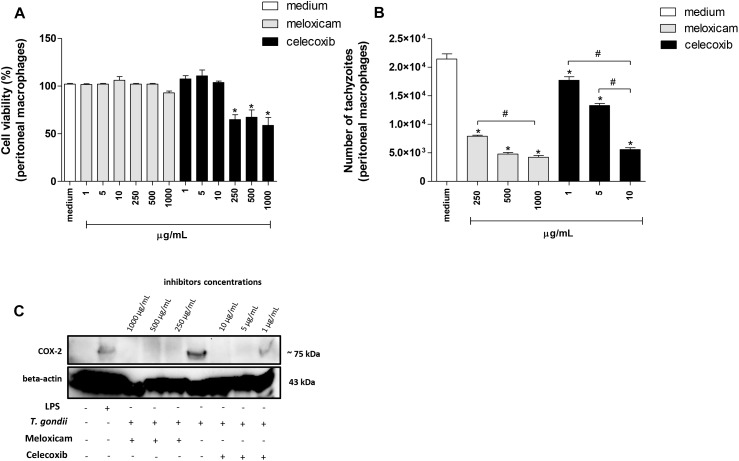
Cell viability, number of tachyzoites and COX-2 expression in *C. callosus*-peritoneal macrophages treated with COX-2 inhibitors. Peritoneal macrophages were seeded in 96-well plates (1 × 10^5^ cells/200 μL) in RPMI medium at 37°C and 5% CO_2_. After 24 h, the cells were treated or untreated with meloxicam or celecoxib in several concentrations for an additional 24 h and submitted to MTT assay **(A)**. Data are expressed as percentage of viable cells (% cell viability) in comparison to untreated cells (100% of cell viability). Data are shown as mean ± SEM from two independents experiments with six replicates **(A)**. In parallel, peritoneal macrophages were seeded in 96-well plates (1 × 10^5^ cells/200 μL) for 24 h, infected with *T. gondii* tachyzoites (2F1 clone, RH strain), treated or untreated with meloxicam or celecoxib for additional 24 h and submitted to *T. gondii* intracellular proliferation assay by β-galactosidase colorimetric reaction **(B)**. Data are shown as mean ± SEM of the number of tachyzoites from three independents experiments with six replicates **(B)**. Differences between groups were analyzed by one-way ANOVA with the Bonferroni multiple comparison *post hoc* test. Significant differences in relation to untreated/uninfected (medium, **A**) or untreated/infected cells (medium, **B**) (^∗^*P* < 0.05), and different concentrations of COX-2 inhibitors (^#^*P* < 0.05). **(C)** Peritoneal macrophages (1 × 10^6^ cells/2000 μL in six-well plates) were infected or not and treated or not with meloxicam or celecoxib in RPMI medium during 24 h. As positive control, peritoneal macrophages were not infected and only treated with 1 μg/mL LPS. Then, macrophages were lysed and submitted to Western blotting for COX-2 and beta-actin detections.

Firstly, we performed MTT assay to verify possible cytotoxicity in peritoneal macrophages treated with meloxicam or celecoxib. The treatment with meloxicam did not induce toxicity, since the cell viability was preserved in all concentrations used in comparison to untreated cells (medium) (Figure 4A). However, at 250, 500, and 1000 μg/mL, celecoxib promoted a significant reduction in cell viability in relation to untreated macrophages (*P* < 0.0001) (Figure 4A).

Next, we performed experiments to verify the *T. gondii* intracellular proliferation in peritoneal macrophages treated or untreated with COX-2 inhibitors using the non-cytotoxic concentrations established by MTT. The lower concentrations of meloxicam (1, 5, and 10 μg/mL) changed the number of tachyzoites slightly compared to untreated macrophages (data not shown). However, 250, 500, and 1000 μg/mL meloxicam and 1, 5, and 10 μg/mL celecoxib in peritoneal macrophages promoted a significant reduction in the number of tachyzoites in comparison to untreated cells (*P* < 0.0001) (Figure 4B). In addition, the reduction in the number of tachyzoites in macrophages treated with celecoxib or meloxicam was dose-dependent, since higher doses of COX-2 inhibitors triggered the best effect on *T. gondii* control (*P* = 0.0008) (Figure 4B). In order to confirm inhibition or reduction of COX-2 expression in cells treated with meloxicam or celecoxib and infected or not by *T. gondii*, Western blotting was performed and it was detected inhibition of COX-2 in all treatments with meloxicam and 5 and 10 μg/mL celecoxib when compared to untreated and infected cells or uninfected LPS-treated cells (Figure 4C). The treatment with 1 μg/mL celecoxib induced only a reduction of COX-2 expression in relation to infected/untreated cells, although this concentration still induced lower parasitism in peritoneal macrophage (Figure 4B,C).

Thus, it is possible to conclude that COX-2 is an important mediator during the *T. gondii* infection triggered by highly virulent strain in rodent cells.

### COX-2 Inhibitors Upregulated TNF and Nitrite in Peritoneal Macrophages Infected With *T. gondii* RH Strain

**FIGURE 5:**
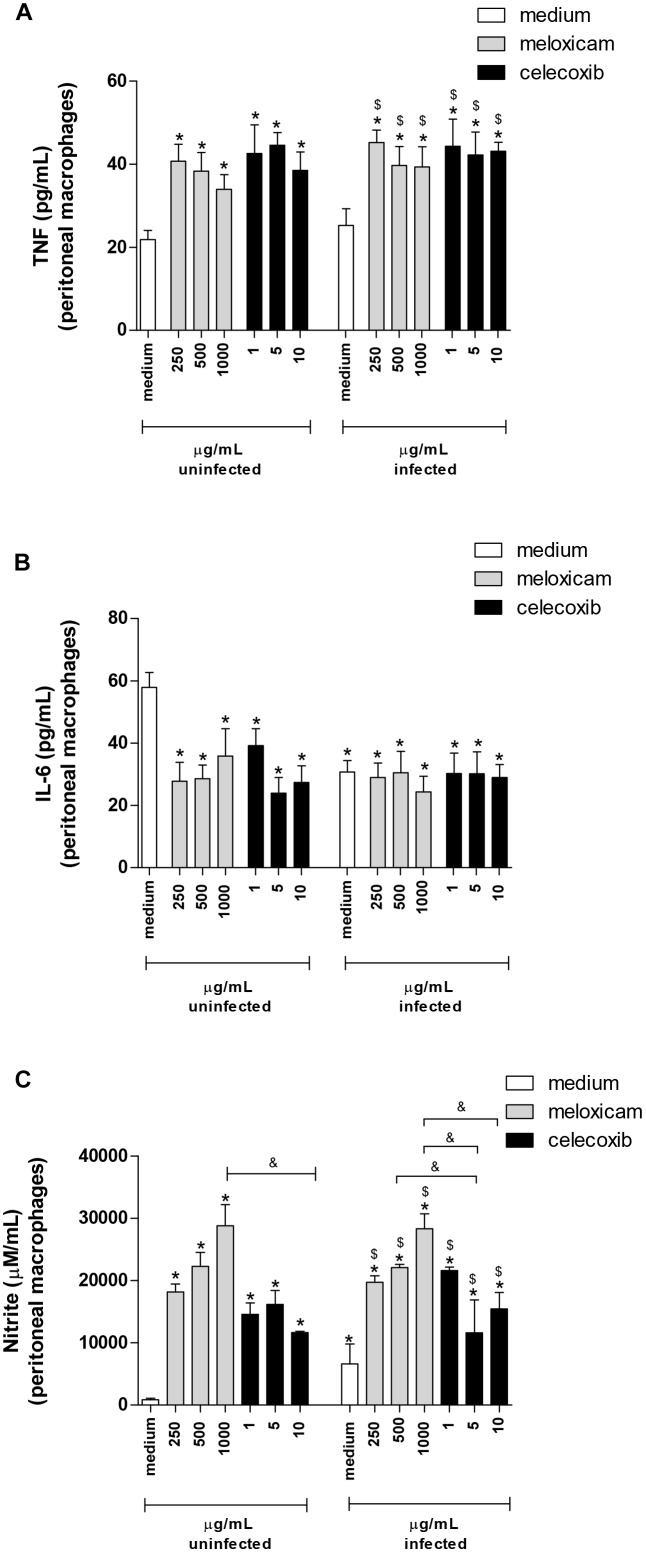
Cytokine and nitrite production in *C. callosus*-peritoneal macrophages treated with COX-2 inhibitors. Peritoneal macrophages were seeded in 96-well plates (1 × 10^5^ cells/200 μL) for 24 h, infected or not with *T. gondii* tachyzoites (2F1 clone, RH strain), treated or untreated with meloxicam or celecoxib for additional 24 h and the supernatants collected for measurement of TNF **(A)** and IL-6 **(B)** by sandwich ELISA, or nitrite **(C)** by the Griess method. Data are shown as mean ± SEM from three independent experiments with six replicates. Differences between groups were analyzed by two-way ANOVA with the Bonferroni *post hoc* test. Significant differences in relation to untreated/uninfected cells (medium) (^∗^*P* < 0.05), untreated/infected cells (medium) (^$^*P* < 0.05), and different concentrations of COX-2 inhibitors regardless of infection (^&^*P* < 0.05).

After evaluating the parasite intracellular proliferation in peritoneal macrophages of *C. callosus*, we verified the cytokine levels in the supernatant of these cells treated or untreated with COX-2 inhibitors (Figure 5).

Macrophages infected and untreated did not alter TNF release in comparison to untreated and uninfected cells (Figure 5A). However, meloxicam and celecoxib significantly upregulated TNF levels, regardless of infection by *T. gondii*, in comparison to uninfected and untreated (*P* = 0.012) or to infected and untreated macrophages (*P* = 0.015) (Figure 5A). In relation to IL-6, infected and untreated macrophages diminished the production of this cytokine in relation to uninfected and untreated cells (*P* = 0.0291) (Figure 5B). Interestingly, both COX-2 inhibitors downmodulated IL-6 secretion regardless of infection compared to uninfected and untreated cells (*P* = 0.029) (Figure 5B), however in the presence of *T. gondii*, COX-2 inhibitors did not change the levels of IL-6 in comparison to untreated and infected cells (Figure 5B). IFN-γ, IL-17A, and IL-10 were undetected in the supernatant of peritoneal macrophages in any conditions (data not shown).

When we investigated nitrite production, there was an increase in production in infected and untreated macrophages in relation to uninfected and untreated cells (*P* < 0.0001) (Figure 5C). Furthermore, all COX-2 inhibitors significantly upregulated nitrite release, regardless of infection, in comparison to uninfected and untreated or to infected and untreated macrophages (*P* < 0.0001) (Figure 5C). Also, the higher doses of meloxicam triggered increased nitrite levels when compared to all doses of celecoxib (*P* < 0.0001) (Figure 5C).

### COX-2 Inhibitors Reduced the *T. gondii* Intracellular Proliferation and PGE_2_ Restored the Parasite Growth in THP-1 Cells Infected With RH Strain

After to verify the role of COX-2 during *T. gondii* infection in rodent cells and tissues, we also observed the function of COX-2 in human cells. For this purpose, we performed the same experiments with human monocyte cells (THP-1 cell line) using only one concentration of meloxicam (250 μg/mL) or celecoxib (10 μg/mL). Firstly, we verified the cell viability in THP-1 cells treated with meloxicam or celecoxib and no significant change was detected (data not shown), as observed for peritoneal macrophage.

**FIGURE 6:**
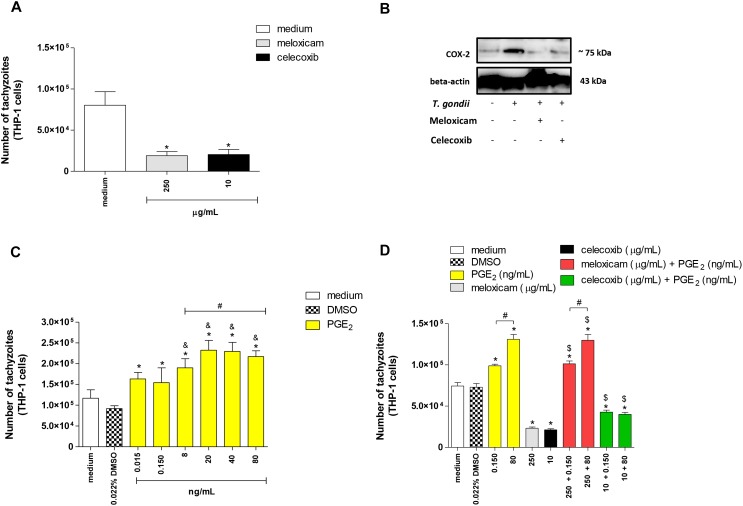
Number of tachyzoites and COX-2 expression in THP-1 cells treated with COX-2 inhibitors and/or PGE_2_. THP-1 cells were seeded in 96-well plates (3 × 10^4^ cells/200 μL) for 24 h, infected with *T. gondii* tachyzoites (2F1 clone, RH strain), treated or untreated with meloxicam or celecoxib for additional 24 h, and submitted to *T. gondii* intracellular proliferation assay by β-galactosidase colorimetric reaction **(A)**. In parallel, THP-1 cells (1 × 10^6^ cells/2000 μL in 6-well plates) were infected or not and treated or not with meloxicam or celecoxib in RPMI medium during 24 h. Then, THP-1 cells were lysed and submitted to Western blotting for COX-2 and beta-actin detections **(B)**. After, THP-1 cells (3 × 10^4^ cells/200 μL in 96-well plates) were infected (2F1 clone) and treated with several concentrations of PGE_2_ for additional 24 h **(C)**, or THP-1 cells were infected and treated with meloxicam or celecoxib plus PGE_2_ for 24 h **(D)**, and all conditions submitted to *T. gondii* intracellular proliferation assay by β-galactosidase colorimetric reaction. DMSO (0.022%) was used to exclude any effect of DMSO in the parasitism. Data are shown as mean ± SEM of the number of tachyzoites from two independents experiments with eight replicates. Differences between groups were analyzed by one-way ANOVA with the Bonferroni multiple comparison *post hoc* test. Significant differences in relation to untreated/infected cells (medium) or infected/DMSO-treated cells (^∗^*P* < 0.05, **A,C,D**), between 0.015 or 0.150 ng/mL and 8–80 ng/mL PGE_2_ (^&^*P* < 0.05, **C**), between different concentrations of PGE_2_ or meloxicam plus PGE_2_ (^#^*P* < 0.05, **C,D**), and between COX-2 inhibitors plus PGE_2_ and respective control (infected and meloxicam or celecoxib-treated cells) (^$^*P* < 0.05, **D**).

Next, we investigated the *T. gondii* intracellular proliferation in THP-1 cells. Both COX-2 inhibitors reduced significantly the number of tachyzoites in comparison to untreated cells (*P* < 0.0001) (Figure 6A). After, we confirmed the COX-2 inhibition in THP-1 cells treated with meloxicam or celecoxib by Western blotting. As presented in Figure 6B, uninfected and untreated THP-1 cells demonstrated basal level of COX-2 expression, while infected and untreated cells showed upregulation of COX-2 in relation to respective control. Furthermore, infected THP-1 cells treated with meloxicam or celecoxib showed reduced COX-2 expression when compared to untreated/infected cells (Figure 6B).

In a second step of the experiments with human cells, we investigated if the role of COX-2 during infection with *T. gondii* in THP-1 cells was dependent of PGE_2_. Then, we treated THP-1 cells with several concentrations of PGE_2_ and it was detected increased number of tachyzoites in relation to infected and untreated cells (medium) (*P* < 0.0001) or infected and DMSO-treated cells (*P* < 0.0001) (Figure 6C). In addition, we verified that higher concentrations of PGE_2_ (8 to 80 ng/mL) and doses from 20 to 80 ng/mL triggered greater number of tachyzoites if compared to 0.015 and 0.150 ng/mL (*P* < 0.0001) or 8 ng/mL (*P* < 0.0001), respectively (Figure 6C). Finally, we blocked COX-2 expression and restored PGE_2_ when added exogenous levels of this lipid mediator in cells treated with meloxicam or celecoxib. As observed in Figure 6A,C, 0.150 and 80 ng/mL PGE_2_ increased the number of tachyzoites (*P* < 0.0001) in dose-dependent manner (*P* < 0.0001) and meloxicam or celecoxib reduced the parasitism (*P* < 0.0001) in THP-1 cells in comparison to untreated cells or DMSO-treated cells (Figure 6D). However, when exogenous levels of PGE_2_ were added in THP-1 cells treated with COX-2 inhibitors, there was a reversion in the effect of meloxicam or celecoxib, since higher parasite intracellular proliferation was detected when compared to untreated cells (*P* < 0.0001), DMSO-treated cells (*P* < 0.0001) and meloxicam (*P* < 0.0001) or celecoxib-treated cells (*P* < 0.01) (Figure 6D).

Thus, it is possible to conclude that COX-2 is an important mediator during the *T. gondii* infection triggered by highly virulent strain in human host cells, and this effect is associated with PGE_2_.

### COX-2 Inhibitors Upregulated MIF, TNF, and Nitrite and Downmodulated IL-8 and IL-10 in THP-1 Cells Infected With *T. gondii* RH Strain

**FIGURE 7:**
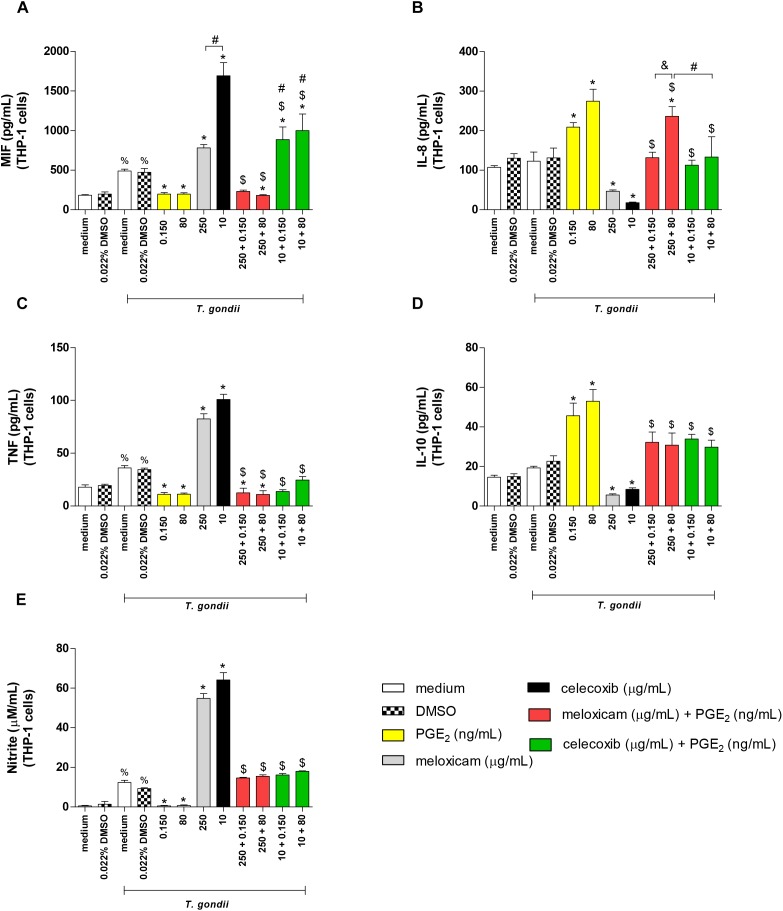
Cytokine and nitrite production in infected THP-1 cells treated with COX-2 inhibitors and/or PGE_2_. THP-1 cells were seeded in 96-well plates (3 × 10^4^ cells/200 μL) for 24 h, infected with *T. gondii* tachyzoites (2F1 clone, RH strain), and treated or untreated with meloxicam or celecoxib in the presence or absence of PGE_2_ for additional 24 h. The supernatants were collected for measurement of MIF **(A)**, IL-8 **(B)**, TNF **(C)**, and IL-10 **(D)** by sandwich ELISA, or nitrite **(E)** by the Griess method. DMSO (0.022%) was used to exclude any effect of DMSO in the cytokine or nitrite production. Data are shown as mean ± SEM from two independents experiments with eight replicates. Differences between groups were analyzed by two-way ANOVA with the Bonferroni *post hoc* test. Significant differences in relation to untreated/uninfected cells (medium) or uninfected/DMSO-treated cells (^%^*P* < 0.05), between untreated/infected cells (medium) or infected/DMSO-treated cells (^∗^*P* < 0.05), between different concentrations of COX-2 inhibitors in the absence or presence of PGE_2_ (^#^*P* < 0.05), between different concentrations of meloxicam plus PGE_2_ (^&^*P* < 0.05), and between COX-2 inhibitors plus PGE_2_ and respective control (infected and meloxicam or celecoxib-treated cells) (^$^*P* < 0.05).

After evaluating the parasite intracellular proliferation in THP-1 cells, we verified the cytokine levels in the supernatant of these cells treated or untreated with COX-2 inhibitors and/or exogenous concentrations of PGE_2_ (Figure 7). The treatment with only DMSO (0.022%) did not change the cytokine profile in relation to untreated cells (medium), regardless of infection with *T. gondii* (Figure 7A–E).

Infected and untreated THP-1 cells augmented MIF (*P* < 0.05), TNF (*P* = 0.0005) and nitrite (*P* < 0.0001) production when compared to uninfected and untreated cells (Figure 7A,C,E). However, in the presence of *T. gondii*, PGE_2_-treated cells reduced significantly the MIF (*P* < 0.05), TNF (*P* < 0.01) and nitrite (*P* < 0.0001) release, while meloxicam and celecoxib increased these productions (*P* < 0.0001) if compared to infected and untreated cells (Figure 7A,C,E). Additionally, celecoxib induced higher MIF secretion in comparison to cells treated with meloxicam (*P* < 0.0001) (Figure 7A). Interestingly, when infected THP-1 cells were treated with COX-2 inhibitor (meloxicam or celecoxib) and PGE_2_, regardless PGE_2_ dose, the MIF (*P* < 0.0001), TNF (*P* < 0.0001) and nitrite (*P* < 0.0001) productions were significantly reduced when compared to respective control (infected and meloxicam or celecoxib-treated cells) (Figure 7A,C,E), although the treatment of infected cells with celecoxib plus PGE_2_ induced more MIF secretion in relation to infected cells and treated with meloxicam plus PGE_2_ (*P* < 0.0001) or infected and untreated cells (*P* < 0.0001) (Figure 7A). Also, infected cells treated with meloxicam plus PGE_2_ reduced the MIF (*P* < 0.05) and TNF (*P* < 0.0001) production in relation to untreated and infected cells (Figure 7A,C).

In relation to IL-8 and IL-10, infected THP-1 cells treated with PGE_2_ increased IL-8 (*P* < 0.0001) and IL-10 (*P* < 0.0001) production, while meloxicam (*P* < 0.05) and celecoxib (*P* < 0.05) reduced it when compared to untreated and infected cells (Figure 7B,D). However, the double treatment of infected cells with COX-2 inhibitors and exogenous PGE_2_ increased IL-8 (*P* < 0.0001) and IL-10 (*P* < 0.0001) release in comparison to respective control (infected and meloxicam or celecoxib-treated cells) (Figure 7B,D). Furthermore, infected cells treated with meloxicam plus 80 ng/mL PGE_2_ produced more IL-8 than meloxicam plus 0.150 ng/mL PGE_2_ (*P* < 0.001) or infected and untreated cells (*P* < 0.001) or celecoxib plus 80 ng/mL PGE_2_ (*P* < 0.001) (Figure 7B). IFN-γ, IL-6 and TGF-β1 did not show significant differences in THP-1 cells under any condition (data not shown).

We also measured cytokines and nitrite in THP-1 cells treated with meloxicam, celecoxib, and PGE_2_ in the absence of *T. gondii* infection in order to verify the effect of all treatments without interference of the parasite. Again, the treatment with DMSO (0.022%) did not change the cytokine profile in THP-1 cells (Supplementary Figures 1A–E). As observed for infected THP-1 cells, meloxicam and celecoxib triggered higher levels of MIF (*P* < 0.0001), TNF (*P* < 0.0001) and nitrite (*P* < 0.0001) when compared to untreated cells (Supplementary Figures 1A,C,E), while the treatment with PGE_2_ in uninfected cells induced only reduction of TNF in relation to untreated cells (*P* < 0.05) (Supplementary Figure 1C) and did not alter the levels of MIF or nitrite (Supplementary Figures 1A,E). When THP-1 cells were blocked to COX-2 expression with meloxicam or celecoxib and treated with PGE_2_, the levels of MIF (*P* < 0.0001), TNF (*P* < 0.0001) and nitrite (*P* < 0.05) were downmodulated in comparison to respective control (uninfected and meloxicam or celecoxib-treated cells) (Supplementary Figures 1A,C,E) or to untreated condition for TNF in cells treated with meloxicam plus PGE_2_ (*P* < 0.05) (Supplementary Figure 1C), except for nitrite in cells treated with meloxicam plus 0.150 ng/mL PGE_2_ (Supplementary Figure 1E). The nitrite levels were upregulated in THP-1 cells treated with meloxicam plus 80 ng/mL PGE_2_ and celecoxib plus PGE_2_ in relation to untreated cells (*P* < 0.0001), but the levels were still lower if compared to respective control (*P* < 0.05) (Supplementary Figure 1E).

Regarding to IL-8 and IL-10 in uninfected THP-1 cells, all doses of PGE_2_ triggered upregulation of these cytokines when compared to untreated cells (*P* < 0.0001) (Supplementary Figures 1B,D). Only celecoxib was able to downmodulate IL-10 in relation to untreated cells (*P* < 0.05) (Supplementary Figure 1D), however the double treatment with any COX-2 inhibitor plus PGE_2_ induced increased IL-8 and IL-10 production in comparison to respective control (uninfected and meloxicam or celecoxib-treated cells) (*P* < 0.01) or untreated cells (*P* < 0.01) (Supplementary Figures 1B,D), except for IL-8 in THP-1 treated with celecoxib plus 0.150 ng/mL PGE_2_ in relation to untreated cells (Supplementary Figure 1B).

### Actions Triggered by COX-2 Inhibitors During Infection by *T. gondii* With Moderately (ME49) and Highly Virulent (RH) Strains

**FIGURE 8:**
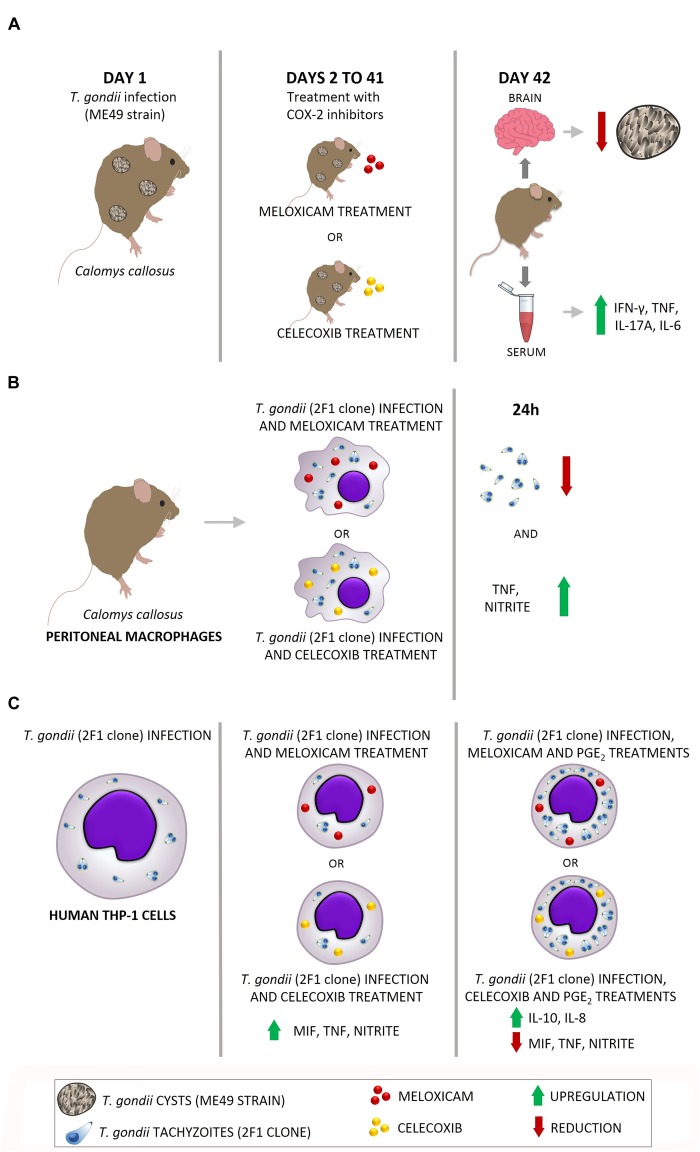
Proposed model of actions induced by COX-2 inhibitors in *in vivo* and *in vitro* experimental models. **(A)** *C. callosus* infected with *T. gondii* (ME49 strain) and treated with COX-2 inhibitors presented reduced parasite burden in brain and upregulation of pro-inflammatory cytokines (IFN-γ, TNF, IL-17A, and IL-6). **(B)** *C. callosus*-peritoneal macrophages treated with COX-2 inhibitors and infected with *T. gondii* (RH strain) demonstrated a reduced parasite intracellular proliferation and upregulation of pro-inflammatory mediators (TNF and nitrite). **(C)** THP-1 cells treated with COX-2 inhibitors and infected with *T. gondii* (RH strain) (middle panel) presented reduced parasite proliferation and upregulation of pro-inflammatory mediators (MIF, TNF, and nitrite) in comparison to untreated/infected cells (left panel); while THP-1 cells treated with COX-2 inhibitors plus exogenous levels of PGE_2_ (right panel) demonstrated increased parasitism, upregulation of IL-10 and IL-8, and downmodulation of pro-inflammatory mediators (MIF, TNF, and nitrite).

A proposed scheme of the actions induced by COX-2 inhibitors in *in vivo* and *in vitro* experimental models is shown in Figure 8. When *C. callosus* females were infected with *T. gondii* (ME49 strain) and treated with COX-2 inhibitors for 40 days, the brains of these animals presented reduced parasite burden, while the serum showed upregulation of pro-inflammatory cytokines (IFN-γ, TNF, IL-17A, and IL-6) (Figure 8A). In addition, when peritoneal macrophages from *C. callosus* females were treated with COX-2 inhibitors and infected with *T. gondii* (2F1 clone, RH strain) for 24 h, the cells demonstrated a lower number of tachyzoites, indicating a reduced parasite intracellular proliferation, and supernatants showed an upregulation of pro-inflammatory mediators (TNF and nitrite) (Figure 8B). When THP-1 cells were infected (2F1 clone) and treated with COX-2 inhibitors during 24 h (middle panel), there was a reduced number of tachyzoites and an upregulation of pro-inflammatory cytokines (MIF, TNF) and nitrite in comparison to untreated/infected cells (left panel) (Figure 8C). Finally, THP-1 cells treated with COX-2 inhibitors plus exogenous levels of PGE_2_ (right panel) demonstrated upregulation of IL-10 and IL-8, and downmodulation of pro-inflammatory mediators (MIF, TNF, and nitrite) in relation to untreated/infected (left panel) or only meloxicam, celecoxib-treated cells (middle panel) (Figure 8C). Thus, it is plausible to conclude that COX-2 is an important mediator that favor *T. gondii* replication, since its blockage triggers significant control of parasitism in rodent and human cells by upregulating important pro-inflammatory mediators involved in the immune response against this parasite. The exogenous PGE_2_ restored the PGE_2_ in THP-1 cells blocked to COX-2 and, consequently, induced higher parasitism by reducing pro-inflammatory mediators and increasing anti-inflammatory mediators (IL-10).

## Discussion

Prostaglandins are lipid mediators able to regulate many functions during an inflammation, such as cytokine production and cellular activation/maturation (Nagamatsu and Schust, 2010; Kalinski, 2012). The production of PGE_2_ occurs when arachidonic acid is converted into prostaglandins by enzymes called cyclooxygenases, as previously described (Batlouni, 2010; Agard et al., 2013; Sharma et al., 2017; Martínez-Colón and Moore, 2018). Many studies demonstrate the role of COX-2 and PGE_2_ during infections triggered by pathogens (Michelin et al., 2005; Abdalla et al., 2008; Tatakihara et al., 2008; Moraes et al., 2015), however there are no studies demonstrating association between COX-2 and susceptibility to *T. gondii* infection. Thus, the aim of the present study was to investigate the role of COX-2 during infection by *T. gondii* in *in vivo* and *in vitro* experimental models. The findings obtained can stimulate study into new therapeutic targets to prevent or treat the acquired toxoplasmosis, a severe public health problem in many countries (Dubey et al., 2012).

Firstly, we verified body-weight change and morbidity scores in females infected with *T. gondii* and treated or untreated with COX-2 inhibitors. In general, infected and COX-2 inhibitor-treated females presented higher body-weight change and lower morbidity score in comparison to infected and untreated females. Some studies demonstrated that the use of non-steroidal anti-inflammatories has been associated with upper gastrointestinal complications (Massó et al., 2010; Castellsague et al., 2012) and cardiovascular and cerebrovascular risk (Fanelli et al., 2017). Thus, it is possible that the meloxicam and celecoxib used to treat the animals triggered blocking of COX-2 and, at the same time, gastrointestinal disorders, promoting body weight loss. On the other hand, the differences between morbidity score in treated and untreated females can be associated to the parasite burden, since inhibitor-treated animals showed reduced tissue parasitism and lower morbidity compared to untreated animals. Then, we can conclude that high parasite burden is proportional to high morbidity score, leading to the symptoms observed in the untreated females.

Next, we observed that meloxicam and celecoxib significantly reduced brain parasitism during the chronic phase of infection triggered by ME49-moderately virulent strain. Additionally, we performed experiments with RH-highly virulent strain in order to verify whether the same phenomena could be repeated. For this purpose, we used as *in vitro* experimental model peritoneal macrophages of *C. callosus* females and human monocyte cells (THP-1 cell line) in order to determine if the role of COX-2 during infection by *T. gondii* would be the same in two different cell types. We verified that peritoneal macrophages and THP-1 cells infected with *T. gondii* (RH strain – 2F1 clone) and treated with meloxicam or celecoxib also reduced significantly the parasite burden, since a lower number of tachyzoites was observed in these cells. Therefore, all COX-2 inhibitors are able to dampen the *T. gondii* proliferation *in vivo* and *in vitro* conditions, regardless of strain or cell types.

Many studies are in agreement with our findings, especially using *T. cruzi* as pathogen. As previously described, mice infected with *T. cruzi* showed reduced parasitism in blood and cardiac muscle when treated with COX-2 inhibitors (meloxicam, etoricoxib, sodium salicylate, aspirin, or celecoxib) (Michelin et al., 2005; Abdalla et al., 2008; Tatakihara et al., 2008). However, Guerrero et al. (2015) did not verify significant changes in the number of *T. cruzi* parasites in cardiac muscle, although the authors have demonstrated that knockout mice for COX-2 showed 30% reduction in blood parasite numbers. Also, COX inhibitors diminished the internalization of *T. cruzi* in mouse peritoneal macrophages (Malvezi et al., 2014) and human monocytes (Carvalho de Freitas et al., 2017), and COX-2 and PGE_2_ are mentioned as inductors of the immunosuppression observed during the acute phase of Chagas disease, favoring the persistence of the parasite in host cells (Pinge-Filho et al., 1999). Additionally, a study has demonstrated that curcumin, a natural product isolated from *Curcuma longa* L. (Zingiberaceae), enhanced survival and provoked a relevant inflammatory response in the heart of mice infected with *T. cruzi*, since curcumin was able to block COX-2 induction and PGE_2_ release (Hernández et al., 2016). A recently published study showed that celecoxib significantly inhibited *T. cruzi* infection in dendritic cell-enriched peripheral human blood mononuclear cell populations, but aspirin, a non-selective COX-1 and COX-2 inhibitor, did not present the same effect (Lonien et al., 2017). The effect of celecoxib in reducing the *T. cruzi* invasion in human dendritic cells was dose-dependent (Lonien et al., 2017), according to our findings in *C. calomys* peritoneal macrophages. Previous studies also demonstrated association between PGE_2_/COX-2 axis and susceptibility to *Leishmania* protozoa. A murine mononuclear phagocyte derived from B-1 cells treated with COX-2 inhibitors and infected with *L. major* presented lower numbers of intracellular amastigotes and reduced PGE_2_ and IL-10 release (Arcanjo et al., 2015), and macrophages infected with *L. donovani* induced COX-2 expression in a toll like receptor-2 (TLR2)-dependent-manner, favoring the infection in these cell types (Bhattacharjee et al., 2016). Thus, it is clear that COX-2 and PGE_2_ can favor the persistence of *T. cruzi* and *Leishmania* in the host.

However, just a small number of studies have verified that COX-2 is induced by *T. gondii* infection, and none of these studies demonstrated that COX-2 is directly involved with *Toxoplasma* susceptibility. As previously described, *T. gondii* was associated to high levels of PGE_2_ and increased COX-2 expression in mouse peritoneal macrophage or skeletal muscle cells (Gomes et al., 2014; Mota et al., 2014). In addition, human epithelial uterine cells infected with *T. gondii* also showed enhanced COX-2 and PGE_2_ expression, although no association was observed in relation to parasite burden (Kim et al., 2006). Thus, our present study is the first to show the role of COX-2 during *T. gondii* infection with moderately or highly virulent strains.

Considering that PGE_2_ is a lipid mediator derived from arachidonic acid by action of COX-2 (Martínez-Colón and Moore, 2018), and that COX-2 inhibitors (meloxicam and celecoxib) reduced *T. gondii* proliferation in peritoneal macrophages and THP-1 cells, we investigated the effect of PGE_2_ restoration in THP-1 cells treated with meloxicam or celecoxib, in order to prove that the role of COX-2 during *T. gondii* infection is dependent of this lipid mediator. As result, it was observed the reestablishment of *T. gondii* growth in THP-1 cells blocked for COX-2 and treated with PGE_2_. Therefore, the role of COX-2 during *T. gondii* infection is PGE_2_-dependent. Our findings are in agreement with Lonien et al. (2017). Human dendritic cells infected by *T. cruzi* and treated with celecoxib plus PGE_2_ presented increased internalization of trypomastigotes in comparison to cells treated with only celecoxib, indicating that PGE_2_ inhibition using celecoxib was reverted and restored the invasion of the parasite in the host cells (Lonien et al., 2017). Also, our previous study showed that human trophoblast cells treated with PGE_2_ presented higher susceptibility to *T. gondii* infection (RH strain), evidencing that PGE_2_ can facilitate parasite proliferation (Barbosa et al., 2014). Additionally, it was demonstrated that annexin A1 decreased parasitism rate in human placenta infected by *T. gondii* and it was associated with reduced levels of PGE_2_ and COX-2 (Oliveira-Cardoso et al., 2018). Thus, to control the parasite replication, the host cells should reduce COX-2 expression to dampen the *T. gondii* susceptibility. In this sense, it was demonstrated that mice microglia cells (BV-2 cells) infected with RH or ME49 strains downmodulated significantly COX-2 expression to control the infection (Hwang et al., 2018). Furthermore, recent studies demonstrated that C57BL/6 mice treated with *T. cruzi* strain Y extracellular vesicle (EV Y) induced increased cardiac parasitism and elevated parasite internalization in bone marrow-derived macrophages, and these effects were associated to lipid body formation and PGE_2_ production, evidencing the role of lipid bodies and PGE_2_ in favor the parasite growth (Lovo-Martins et al., 2018). In addition, the treatment with drugs as NS-398 and aspirin inhibited PGE_2_ release and reduced significantly *T. cruzi* replication in macrophages, demonstrating eicosanoids as a parasite escape mechanism (Almeida et al., 2018). Then, our study is the first to show the potential effect of COX-2 in favoring the replication of *T. gondii* in the host cells, regardless of strain or cell types (rodent and human cells), becoming *C. callosus* an excellent model to study the role of COX-2 during infections by pathogens, as *T. gondii*.

After verifying that COX-2 inhibitors decreased the susceptibility to *T. gondii*, we investigated the immune mechanisms that triggered these findings. Infected *C. callosus* treated with meloxicam or celecoxib upregulated IFN-γ, IL-17A, TNF and IL-6, while peritoneal macrophages or THP-1 cells treated with both COX-2 inhibitors upregulated TNF and nitrite, or TNF, MIF and nitrite, respectively. In the absence of infection, peritoneal macrophages and THP-1 cells demonstrated similar findings, since basal levels of COX-2 are observed in these cells and the inhibition of this basal expression seems to be sufficient to trigger a pro-inflammatory response. Thus, it is possible conclude that meloxicam and celecoxib induce a pro-inflammatory immune response, which triggers significant reduction in *T. gondii* infection. Many studies demonstrated the important protective role of IFN-γ, nitrite (Gazzinelli et al., 1994; Lang et al., 2007; Kemp et al., 2013; Koblansky et al., 2013; Behnke et al., 2017; Abreu-Cabral et al., 2018), IL-17A (Kelly et al., 2005), MIF (Ferro et al., 2008; Flores et al., 2008; Terrazas et al., 2010; Gomes et al., 2011, 2018; Barbosa et al., 2014), TNF and IL-6 (Castro et al., 2013; Barbosa et al., 2014, 2015) during infection by *T. gondii*. Our recent studies showed that IL-6, TNF and MIF are the most important cytokines involved in the immune response to *T. gondii* in human trophoblast cells, human explants from third trimester and murine maternal-fetal interface, allowing a significant reduction in the vertical transmission of the parasite (Barbosa et al., 2015; Silva et al., 2017; Gomes et al., 2018). Oliveira et al. (2010) observed higher IFN-γ and IL-2 concentrations in serum of rats infected with *T. cruzi* and treated with meloxicam. Also, increased nitrite production and high numbers of inflammatory infiltrates was observed in the colon of mice infected with *T. cruzi* and treated with COX-2 inhibitor, regardless of time of infection, or acute or chronic phase (Oda et al., 2017). Additionally, upregulation of IL-1β, nitrite, IL-12, and IL-10 was verified in murine peritoneal macrophages (Malvezi et al., 2014) and human monocytes (Carvalho de Freitas et al., 2017) infected with *T. cruzi* and treated with aspirin-COX inhibitor. However, Lonien et al. (2017) did not verify TNF, IL-6, IL-8, IL-10, and NO production after celecoxib treatment in human dendritic cells, although the COX-2 inhibitor did control the *T. cruzi* infection in these cells. Therefore, the inflammatory immune response induced by both COX-2 inhibitors can be one of the mechanisms responsible for *T. gondii* control *in vivo* and *in vitro* conditions. Probably, other mechanisms are triggered by COX inhibitors, however future studies are necessary to verify this hypothesis. Furthermore, future experiments are necessary to verify the role of COX-2 during *T. gondii* infection in the maternal-fetal interface in order to verify whether the same findings are observed during congenital toxoplasmosis.

In THP-1 cells, we observed higher levels of MIF and lower levels of IL-8 and IL-10 during meloxicam or celecoxib treatments. IL-10 is an anti-inflammatory cytokine and it is associated with increased *Toxoplasma* replication in many cell types, including human trophoblast and monocyte cells (Barbosa et al., 2008, 2015; Franco et al., 2011; Castro et al., 2013). Therefore, it is possible that the lower IL-10 release in cells blocked to COX-2 helped the parasite control.

IL-8 is a member of the CXC chemokine family produced by macrophages and other cell types, and it is significantly associated to neutrophil chemoattractant and activator, then it is an important mediator during innate immune response to several pathogens and other inflammation types (Dong and Zheng, 2015). For example, IL-8 is predominant in pregnant women with positive IgM *T. gondii* serology (Nejad et al., 2011), and participates of immune response to *Trichomonas vaginalis* (Nam et al., 2012) and visceral leishmaniasis (Frade et al., 2011). Many types of extracellular signals activate intracellular pathways resulting in IL-8 release, and this cytokine has ability to trigger mitogen-activated protein kinase (MAPKs), as extracellular regulating kinase (ERK)1/2, and finally promote inflammation and granulocyte recruitment (Dong and Zheng, 2015). Sommerville et al. (2013) verified that *T. gondii* produces a homolog of MIF, called TgMIF, and this MIF of the parasite was able to induce IL-8 in human peripheral blood mononuclear cells (PBMC) and triggered ERK1/2 in murine bone marrow-derived macrophages. Probably, IL-8 induced by TgMIF can attract neutrophil and immature dendritic cells to the site of infection, promoting a dissemination of the infection by host organism, thus TgMIF may play an important immunomodulatory function to favor the parasite propagation, even recruiting inflammatory cells (Sommerville et al., 2013). Our previous study demonstrated that MIF is able to control *T. gondii* replication only in high doses, since there is absence of ERK1/2 and PGE_2_ production (Barbosa et al., 2014). In the present study, we verified that higher MIF release in THP-1 cells treated with meloxicam or celecoxib contributed to control of the parasitism and, at the same time, reduced IL-8. We can speculate that both decreased IL-8 secretion plus high levels of MIF could trigger lower levels of ERK1/2 phosphorylation in THP-1 cells, and thinking about *in vivo* conditions, all these phenomena may dampen propagation of *T. gondii* for the host organism. Then, it is possible to hypothesize that IL-8 has an important role in favor the dissemination of infection, even recruiting inflammatory cells to site of infection. It can be reinforced when THP-1 cells were infected and treated with PGE_2_: increased IL-8 production and, at the same time, elevated *T. gondii* proliferation. However, future studies are necessary to verify the intracellular pathway triggered by IL-8 in monocytes infected by *Toxoplasma.*

Taken together, we can conclude that COX-2 is an immune mediator involved in the susceptibility to *T. gondii* regardless of strain or cell types, since the inhibition of cyclooxygenases was able to reduce infection in the host cells by triggering a classical and potent pro-inflammatory immune response. Thus, COX-2 can be a potential target for future therapeutic strategies against *T. gondii* infection.

## Author Contributions

BFB designed the experiments. AP, RS, PF, GS, IM, MR, AOG, AR, PG, ER, and BFB performed the experiments. AP, RS, PF, NS, and BFB analyzed the data. BFB, TM, JM, NS, and EF provided the reagents. BFB, PF, TM, JM, NS, and EF discussed the findings. BFB, MR, JM, and EF reviewed the manuscript. All authors approved the final version of the manuscript.

## Conflict of Interest Statement

The authors declare that the research was conducted in the absence of any commercial or financial relationships that could be construed as a potential conflict of interest.
