# Supplementary figures and images for: Cyclooxygenase (COX)-2 Inhibitors Reduce Toxoplasma gondii Infection and Upregulate the Pro-inflammatory Immune Response in Calomys callosus Rodents and Human Monocyte Cell Line
Source: Front Microbiol. 2019 Feb 12;10:225. doi: 10.3389/fmicb.2019.00225 (PMC6379304; doi:10.3389/fmicb.2019.00225)

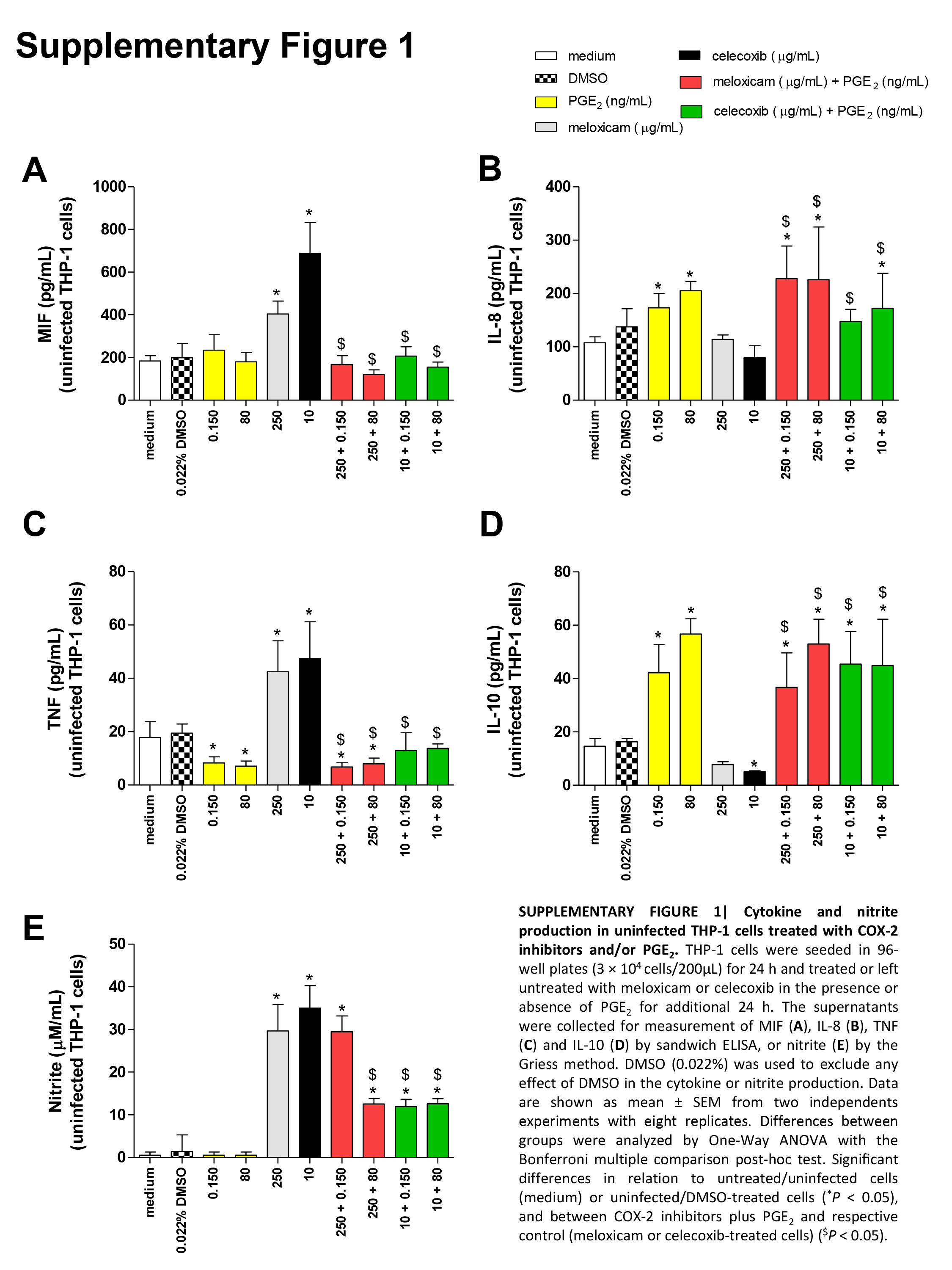

Supplement: Supplementary file 1 [file Image_1.TIF]
